# The development of cognitive control in children with autism spectrum disorder or obsessive-compulsive disorder: A longitudinal fMRI study

**DOI:** 10.1016/j.ynirp.2021.100015

**Published:** 2021-05-30

**Authors:** Bram Gooskens, Dienke J. Bos, Jilly Naaijen, Sophie E.A. Akkermans, Anna Kaiser, Sarah Hohmann, Muriel M.K. Bruchhage, Tobias Banaschewski, Daniel Brandeis, Steven C.R. Williams, David J. Lythgoe, Jan K. Buitelaar, Bob Oranje, Sarah Durston, the TACTICS consortium

**Affiliations:** a Department of Psychiatry, Brain Center, University Medical Center Utrecht, Utrecht University, Utrecht, the Netherlands; b Donders Centre for Cognitive Neuroimaging, Donders Institute for Brain, Cognition and Behaviour, Radboud University, the Netherlands; c Department of Cognitive Neuroscience, Donders Institute of Brain, Cognition and Behaviour, Radboud University Medical Center, Nijmegen, the Netherlands; d Department of Child and Adolescent Psychiatry and Psychotherapy, Central Institute of Mental Health, Medical Faculty, Mannheim/Heidelberg University, Mannheim, Germany; e Department of Neuroimaging, King's College London, Institute of Psychiatry, Psychology and Neuroscience, London, UK; f Department of Child and Adolescent Psychiatry and Psychotherapy, Psychiatric Hospital, University of Zurich, Zurich, Switzerland; g Center for Integrative Human Physiology, University of Zurich, Zurich, Switzerland; h Neuroscience Center Zurich, University of Zurich, Zurich, Switzerland; i ETH Zurich, Zurich, Switzerland; j Karakter Child and Adolescent Psychiatry University Center, Nijmegen, the Netherlands

**Keywords:** Autism spectrum disorder, Obsessive-compulsive disorder, Repetitive behavior, Cognitive control, fMRI

## Abstract

Repetitive behavior is a core symptom of Autism Spectrum Disorder (ASD) and Obsessive-Compulsive Disorder (OCD), and has been associated with impairments in cognitive control. However, it is unclear how cognitive control and associated neural circuitry relate to the development of repetitive behavior in children with these disorders. In a multicenter, longitudinal study (TACTICS; Translational Adolescent and Childhood Therapeutic Interventions in Compulsive Syndromes), the development of cognitive control was assessed during late childhood using a longitudinal fMRI design with a modified stop-signal task in children with ASD or OCD, and typically developing (TD) children (baseline: N = 95 (8-12 y), follow-up: N = 53 (10-14 y), average interval: 1.48 y (sd: 0.36, range: 0.98–2.52 y). Stop-signal reaction time (SSRT) decreased over development, regardless of diagnosis. Repetitive behavior in children with ASD and OCD was not associated with performance on the stop-signal task. There were no whole-brain between-group differences in brain activity, but ROI-analyses showed increases in activity in right precentral gyrus over development for children with OCD. In sum, even though subtle differences were observed in the development of brain activity in children with OCD, overall the findings suggest that the development of cognitive control, as assessed by the stop signal task, is similar to typical in children with ASD and OCD.

## 1 Introduction

Cognitive control is the ability to flexibly adjust behavior in the context of dynamic changing goals. This ability is crucial for successfully navigating the demands of daily life. The development of cognitive control starts in infancy, and continues well into adulthood. (Luna, Padmanabhan & O'Hearn, 2010; Luna et al., 2015). Impairments in cognitive control are thought to play a role in repetitive behavior and the emergence of neurodevelopmental disorders where such behaviors play a role, such as autism spectrum disorders (ASD) and obsessive-compulsive disorder (OCD) (Chamberlain et al., 2005; Hill, 2004; Lipszyc and Schachar, 2010; Moritz et al., 2002; Mosconi et al., 2009; Snyder et al., 2015).

Repetitive behaviors in ASD and OCD show qualitative and quantitative similarities. For instance, restricted interests in ASD and obsessions in OCD both involve persistent repetitive thoughts, whereas stereotyped behavior in ASD and compulsions in OCD both reflect symptoms that manifest as repetitive behavior that must be carried out (Jiujias et al., 2017). Furthermore, children with ASD and OCD have been reported to display similar levels of sameness- and repetitive sensory-motor behaviors (Zandt et al., 2007). Previous studies have demonstrated increased rates of obsessive-compulsive symptoms in ASD and vice versa (Ivarsson and Melin, 2008; Leyfer et al., 2006). However, the purpose of these behaviors or the context in which they take place may differ between the disorders. While repetitive behavior in ASD is typically maintained by reinforcement, in OCD it usually has the purpose of relieving anxiety or distress caused by the obsession (McDougle et al., 1995; Zandt et al., 2007, 2009, ). The extent to which ASD and OCD have distinct versus common neural correlates of repetitive behavior, and how this relates to the development of cognitive control is still unknown.

A core feature of cognitive control is the ability to inhibit prepotent responses. This feature is modeled in the laboratory by the stop-signal task (SST), where the participant is challenged to withhold a motor response after it has been initiated (Logan and Cowan, 1984). Measures derived from this task include the stop-signal reaction time (SSRT), which reflects the speed of the stopping process, and speeds up during typical development (Bedard et al., 2002, Tillman et al., 2008). In OCD, cross-sectional SST studies have suggested that impairments in cognitive control are not as evident in children and adolescents (Gooskens et al., 2019; Hybel et al., 2017a; Marzuki et al., 2020; Rubia et al., 2010; Woolley et al., 2008) as they are in adults with OCD, where longer SSRTs compared to controls have often been reported (Chamberlain et al., 2007; Kang et al., 2013; Penadés et al., 2007; De Wit et al., 2012). Similarly, SSRT has not always been found to differ between children with ASD and typically developing children (Adams and Jarrold, 2012; Gooskens et al., 2019; Ozonoff and Strayer, 1997; Albajara Sáenz et al., 2020; Schmitt et al., 2018). However, in ASD, the developmental trajectory of SST performance seems to be attenuated, so that the stopping ability of children with ASD and typically developing children seem to start diverging at approximately 10 years of age (Schmitt et al., 2018). Even though longitudinal studies are lacking, previous work indicates that the transition from childhood to adulthood represents a period of significant change in the ability to inhibit prepotent responses, such as in the SST. Moreover, previous work in children with OCD has suggested that, even in the absence of case-control differences, cognitive control to be predictive of response to cognitive behavioral therapy (Hybel et al., 2017b), underscoring the clinical relevance of understanding cognitive control development in ASD and OCD.

Most functional Magnetic Resonance Imaging (fMRI) studies on the typical development of cognitive control have found increases in activity of prefrontal cortex (PFC) with development, often related to improvements in task performance (Bunge et al., 2002; Cohen et al., 2010; Durston et al., 2002; Rubia et al., 2007). However, age-related decreases in PFC activity have also been reported (Somerville et al., 2011), and have been suggested to reflect a developmental decrease in the effort required to exert cognitive control (Tamm et al., 2002), consistent with a shift from diffuse to focal engagement of PFC (Durston et al., 2006). Additionally, nonlinear developmental changes, where brain activity in PFC increases from childhood to adolescence, followed by decreases into adulthood have been reported (Luna et al., 2010; Somerville et al., 2011).

fMRI studies using the stop-signal task to compare brain activity in children with OCD to typically developing children have reported hypoactivation in brain regions associated with cognitive control, including in PFC regions such as the dorsolateral prefrontal cortex (DLPFC) and inferior frontal gyrus (IFG), but also striatum and thalamus (Carlisi et al., 2017; Rubia et al., 2010; Woolley et al., 2008). Contrary to hypoactivation observed in children with OCD, hyperactivation in PFC regions including the middle frontal gyrus (MCG) and the IFG has been reported in children with ASD during cognitive control (Albajara Sáenz et al., 2020; Carlisi et al., 2017; Chantiluke et al., 2015). However, our previous work directly comparing ASD and OCD reported no group differences in brain activation during cognitive control compared to typically developing children (Gooskens et al., 2019), and longitudinal work investigating the development of cognitive control and its neurobiological correlates in ASD and OCD is lacking.

In the current study, we recruited children with ASD, OCD and typically developing children (9–14 years) in the context of a multi-center collaborative initiative, the Translational Adolescent and Childhood Therapeutic Interventions in Compulsive Syndromes (TACTICS) consortium. The primary objective of this study was to investigate the development of cognitive control and associated neural circuitry in relation to repetitive behavior in children with ASD and OCD. We operationalized cognitive control as the ability to stop an ongoing response in the context of a stop-signal task during fMRI. At baseline (mean age = 10 years), we found no differences in cognitive control and associated neural circuitry in children with ASD or OCD compared to typically developing (TD) children. However, we did find that increased activity in prefrontal cortex was associated with ADHD symptoms (Gooskens et al., 2019). As cognitive control improves over development, and given that deficits in cognitive control in ASD and OCD may emerge later in development, we performed a follow-up study. We hypothesized that SSRT would decrease over development for all children, indicating improvement in cognitive control. We further hypothesized that developmental improvements in task performance would be associated with increased brain activity in prefrontal brain areas for typically developing children. Finally, we hypothesized that children with OCD and ASD would show delay in the development of cognitive control, reflected by smaller improvements in task performance and an attenuated increase in brain activity in prefrontal brain areas over development compared to TD children, and that these changes in cognitive control would relate to the presence and severity of repetitive behavior.

## 2 Methods

### 2.1 Participants

At baseline, we were able to include data for 122 participants between 8 and 12 years (ASD = 38, OCD = 23, TD = 61) (details are described in Gooskens et al., 2019). Ninety-three participants (ASD = 34, OCD = 15, TD = 44) participated in a follow-up visit. Participants' reasons for drop-out were mainly loss of interest, or wearing dental braces that prevented them from participating in an MRI assessment. Follow-up time was 1.48 years, on average, and ranged from 0.98 to 2.52 years. Participants were seen at the same four sites across Europe (Central Institute of Mental Health, Medical Faculty Mannheim, University of Heidelberg Mannheim, Germany; King's College London, London, United Kingdom; Radboud University Medical Center and the Donders Institute for Brain, Cognition and Behavior, Nijmegen, The Netherlands; University Medical Center Utrecht Brain Center, Utrecht, The Netherlands) and were commissioned by a multicenter study (COMPULS: Naaijen et al., 2016) as part of the overarching TACTICS collaborative initiative (http://www.tactics-project.eu). Exclusion criteria for all participants were an estimated total IQ below 70 and insufficient comprehension and speaking ability of the native language of the country. For MR scanning, the presence of metal objects in the body (i.e., pacemaker, dental braces), neurological illness or other contra-indications for MRI-assessment were exclusion criteria. Participants were asked to abstain from stimulant medication 24 h before scanning. For both diagnostic groups, a concurrent diagnosis of the other disorder was an exclusion criterion (i.e. comorbid OCD for a child with ASD, or vice versa). In TD participants or their first-degree relatives the presence of any psychiatric diagnosis was an exclusion criteria. After description of the study, parents of all participants gave written informed consent.

### 2.2 Phenotyping

Participants with ASD or OCD were diagnosed according to *The Diagnostic and Statistical Manual of Mental Disorders*, 4th edition, Text Revision (American Psychiatric Association, 2000) or 5th edition criteria (American Psychiatric Association, 2013). At baseline, ASD diagnosis was ascertained by a trained psychologist at each site using the Autism Diagnostic Interview-Revised (ADI-R; Lord et al., 1994). The Children's Yale-Brown Obsessive-Compulsive Scale (CY-BOCS; Scahill et al., 1997) was used as a severity scale for obsessions and compulsions for all children with OCD. This interview was repeated at follow-up and also performed in participants with ASD if screening questions suggested the presence of clinically significant obsessions or compulsions.

To determine the presence of possible comorbidities, all parents were interviewed using the structured Diagnostic Interview Schedule for Children (DISC-IV, parent version; Shaffer et al., 2000), the Development and Well-being Assessment (DAWBA; Goodman et al., 2000) or the Kiddie Schedule for Affective Disorders and Schizophrenia (K-SADS; Kaufman et al., 1997) at both baseline and follow-up. Total Intellectual Quotient (IQ) was estimated using a shortened version of the Wechsler Intelligence Scale for Children (WISC-III; (Wechsler, 2002)). At both timepoints, repetitive behavior was assessed using the Repetitive Behavior Scale – Revised (RBS-R; Bodfish et al., 2000). In addition, the Conners’ Parent Rating Scale – Revised (CPRS-R:L; Conners et al., 1998) was used to rate Attention Deficit/Hyperactivity Disorder (ADHD) symptoms at both timepoints.

Information on medication was collected through parental report.

At baseline, four children with ASD were being treated with psychostimulants, two with antipsychotics, one with a combination of both and one child took low-dose naltrexone.

Within the OCD group, seven children were being treated with antidepressants, one with antipsychotics and one with both. In the follow-up sample, five children with ASD were being treated with psychostimulants, one with psychostimulants and antipsychotics, and one with an antipsychotic and an antidepressant. Three children with OCD were being treated with antidepressants, one with an antipsychotic and an antidepressant, and one with a psychostimulant.

At baseline, seven children with ASD had a current comorbid diagnosis of attention deficit hyperactivity disorder (ADHD), another two children had both comorbid ADHD and oppositional defiant disorder (ODD). In the OCD group, two participants had comorbid ADHD. In the follow-up sample, three children with ASD had a current comorbid diagnosis of ADHD, one child had comorbid depressive disorder, and one child had both comorbid ADHD and ODD. In the OCD group, one child had comorbid ADHD.

### 2.3 Stop-signal task

Our modified version of the stop-signal task (Rubia et al., 2003; Gooskens et al., 2019) requires participants withholding a motor response to a go stimulus when it is randomly followed by a stop signal. During go-trials (80% of trials), subjects were instructed to make a button response with their right index- or middle finger corresponding to the arrow direction (left arrow: index finger, right arrow: middle finger, duration arrow: 500 ms). The mean inter-trial interval (ITI) was randomly jittered between 1.6 and 2.0 s to optimize statistical efficiency. During stop trials (20% of trials), go-signals were followed (approximately 250 ms later) by arrows pointing upwards (stop signals), and participants were instructed to withhold (stop) their motor response. The delay between a go- and stop-signal (stop-signal delay: SSD) was dynamically adapted (start: 250 ms, step size: 50 ms) in response to each subject's stop performance using a staircase algorithm. This procedure ensured that the session concluded with an approximately equal number of successful and failed stop-trials. Before scanning, participants performed a brief practice session of the task.

### 2.4 Task performance measures

Our main measure of interest was stop-signal reaction time (SSRT). Additional measures were mean reaction time (MRT) on correct go-trials, mean stop-signal delay (SSD), number of non-responses to go-trials (omissions) and number of incorrect responses to go-trials, where a subject pressed the wrong button (left instead of right, or vice versa) on a go-trial (choice errors). The SSRT was estimated using the integration method from Verbruggen et al., 2013, Verbruggen et al., 2019: first, reaction times (RT) to correct go-trials were rank ordered. Subsequently, the *n*th go-RT was selected, where *n* was derived by multiplying the number of correct go-trials by the probability that one would respond to a stop-signal (*P* (respond | stop-signal)). The SSRT was then estimated by subtracting the mean SSD from the *n*th go-RT.

Baseline data excluded from statistical analysis is described in detail in our earlier paper (Gooskens et al., 2019). Of the 93 participants who participated in the follow-up visit, data from six participants could not be analyzed due to incomplete task performance data (ASD N = 3, TD N = 3). To optimize data quality for statistical analysis, we used Congdon and colleagues’ method (2012) to exclude participants with SSRT values below 50 ms (ASD N = 6, OCD N = 1, TD N = 7) and accuracy below 25% on successful stop-trials (OCD N = 1) (Gooskens et al., 2019). As such, data were available for behavioral analysis for 122 participants at baseline (ASD N = 38, OCD N = 23, TD N = 61) and 72 participants at follow-up (ASD N = 25, OCD N = 13, TD N = 34) (see Table 1, Table 2 for sample characteristics).

**Table 1:** Sample characteristics [full behavioral sample].

	ASD	OCD	TD	Statistical test	*P*-value
*n* T1	38	23	61		
*n* T2	25	13	34		
Sex (m/f) T1	27/11	11/12	33/28	χ^2^ (2) = 4.019	.134
Sex (m/f) T2	16/9	7/6	19/15	χ^2^ (2) = 0.522	.770
Age in years T1, mean (SD)	10.99 (1.28)	10.80 (1.44)	10.76 (1.20)	F_2, 119_ = 0.408	.666
Age in years T2, mean (SD)	12.32 (1.44)	11.84 (1.64)	11.87 (1.24)	F_2, 69_ = 0.912	.406
Interval in years (SD)	1.46 (0.35)	1.49 (0.35)	1.46 (0.34)	F_2, 52_ = 0.034	.967
Estimated IQ T1, mean (SD)	109.21 (15.41)	104.27 (14.66)	110.61 (11.23)	F_2, 119_ = 1.845	.163
Estimated IQ T2, mean (SD)	109.12 (15.01)	104.35 (12.32)	113.67 (9.74)	K–W χ2 (2) = 4.335	.114

Abbreviations: T1, Timepoint 1/baseline; T2, Timepoint 2/follow-up; m, male; f, female; SD, standard deviation.

**Table 2:** Clinical measures and task performance [full behavioral sample].

	T1	T2	T1	T2	T1	T2	Statistics		
	ASD *N* = 38	ASD *N* = 25	OCD *N* = 23	OCD *N* = 13	TD *N* = 61	TD *N* = 34	Time	Group	Time* Group
	Mean (SD)	Mean (SD)	Mean (SD)	Mean (SD)	Mean (SD)	Mean (SD)	*P*-value	*P*-value	*P*-value
**Questionnaires**									
*CY-BOCS*									
- Obsessions			7.00 (5.41)	8.50 (5.41)			.839	n.a.	n.a.
- Compulsions			9.91 (4.02)	8.62 (4.02)			.217	n.a.	n.a.
- Total score			16.48 (7.56)	15.15 (10.23)			.317	n.a.	n.a.
*RBS-Revised*									
- Compulsivity	2.68 (3.39)	2.88 (4.46)	4.65 (3.54)	4.15 (4.31)	0.05 (0.28)	0.09 (0.29)	.210	>.001*^c^	n.s.
- Total score	23.32 (18.90)	19.44 (18.45)	17.52 (13.17)	14.62 (11.23)	0.56 (1.23)	0.79 (1.97)	.864	>.001*^d^	n.s.
*CPRS-Revised: Long*^f^									
- Inattention	62.52 (11.72)	60.24 (10.34)	57.36 (10.61)	56.50 (14.22)	45.83 (6.70)	46.18 (6.05)	.817	>.001*^d^	n.s.
- Hyperactivity	62.90 (13.16)	61.46 (13.62)	61.29 (10.51)	61.17 (16.18)	46.12 (3.99)	46.44 (4.93)	.600	>.001*^d^	n.s.
- Total score	64.40 (12.78)	61.88 (11.77)	59.93 (10.77)	59.08 (14.35)	45.44 (5.11)	45.25 (4.61)	.661	>.001*^d^	n.s.
**Performance**									
MRT	569.82 (79.84)	508.35 (62.91)	551.61 (77.33)	558.34 (104.66)	564.65 (81.91)	546.92 (66.15)	.213	.887	n.s.
SSD	350.94 (97.81)	334.22 (72.70)	317.98 (114.98)	402.17 (98.18)	345.10 (87.95)	352.70 (65.68)	.067	.781	.022*^b^
SSRT	186.30 (82.88)	145.62 (48.74)	211.34 (114.45)	122.75 (54.54)	186.70 (69.20)	164.66 (59.83)	.009*^a^	.964	n.s.
Omissions^g^	5.22	2.35	2.11	1.58	2.26	1.96	.535	.007*^e^	n.s.
Choice errors^g^	4.84	3.94	4.33	3.09	4.29	4.20	.685	.682	n.s.
Successful Stopping^g^	51.45	51.33	50.65	52.56	51.53	51.47	.501	.956	n.s.

Abbreviations: T1 = Timepoint 1; T2 = Timepoint 2; ASD = autism spectrum disorder; OCD = obsessive-compulsive disorder; TD = typically developing group; SD = standard deviation; m/f = male/female; ADI = Autism Diagnostic Interview; CY-BOCS = Children's Yale-Brown Obsessive-Compulsive scale; RBS = Repetitive-Behavior scale; CPRS = Conners' Parent Rating scale; MRT = Mean reaction time; SSD = Stop-signal delay; SSRT = Stop-signal reaction time; n.a. = not applicable; n.s. = not significant (removed from model).

a SSRT decreased over development across all groups.

b SSD increased over development for children with OCD, compared to both children with ASD and TDC.

c OCD > ASD > TD.

d ASD, OCD > TD.

e ASD > TD.

f Displayed scores are based on T-scores.

g Omissions, choice errors and successful stops are displayed in % of total trials.

### 2.5 Behavioral analysis

Behavioral data analysis was performed in SPSS version 25 (IBM) and R-software 3.5.1 (R Core Team, 2019). Baseline group differences in demographic and clinical measures were tested using the appropriate Pearson's χ^2^-tests or one-way analyses of variance (ANOVA). We used Levene's test for homogeneity of variance and Shapiro-Wilk normality tests to check if assumptions of homogeneity of variance and normality were met. If data did not meet these assumptions, we ran non-parametric tests (Kruskal Wallis rank sum test or Mann–Whitney *U* test).

*Full behavioral sample analyses.* To examine whether there were any group differences in the developmental trajectory of task-performance, we used linear mixed-effects (LME) models in the lme4 package in R (Bates et al., 2015). LME models allow for missing data (provided the data is missing at random), which therefore permits the use of all acquired data. For each task performance measure of interest (MRT, SSD, SSRT, number of omission and commission errors) we fit LME models with diagnostic group, time point (1 and 2), and age as fixed factors, and within subject dependence as a repeated measures random factor. Age was included as a fixed factor in all analyses, given the importance of developmental stage when investigating growth trajectories. Given that there were no significant differences in sex between the diagnostic groups, this factor was subsequently left out of the model. Because site is not a systematic and reliable predictor for explaining relationships with other dependent variables, it was only included in the model as a covariate when its effect reached statistical significance. If the diagnostic status by time interaction did not render any significant effects, it was removed from the design. For significant interaction effects, we ran post-hoc pairwise comparisons using least-squares means.

To be sure that no bias was introduced by participants with data at only one timepoint, we ran follow-up analyses that only included subjects who had complete data at both timepoints (see Supplemental Material). In addition, as children with ASD and OCD often have symptoms of ADHD, and to replicate findings from our baseline paper (Gooskens et al., 2019), we repeated the analysis on ADHD symptomatology with subjects who had complete data at both timepoints, and used a median split to create two groups based on CPRS-R score (Low <56; High >57) (see Supplemental Material).

### 2.6 MRI scanner information

At the four different sites, comparable 3-T MRI scanners were used (Siemens Trio and Siemens Prisma, Siemens, Erlangen, Germany; General Electric MR750, GE Medical Systems, Milwaukee, WI, USA; Philips 3 T Achieva, Philips Medical Systems, Best, The Netherlands). We used classic, gradient echo EPI sensitive to BOLD MR contrast (TR: 2070 ms, TE: 35 ms). More detailed information on the scanners and sequences is available in the baseline paper (Gooskens et al., 2019).

Before participating in the MR session, children at each site were prepared for scanning using a simulation session with a mock scanner (except for Mannheim). In this session, children were familiarized with MR sounds, the button box needed for task completion, and lying still in the scanner environment. If a child (or his/her parent) reported enhanced anxiety to enter the MR scanner, the session was ended. This procedure has proven successful in reducing anxiety for the MR session (Durston et al., 2009).

### 2.7 fMRI preprocessing and first-level analysis

fMRI preprocessing was performed using standard procedures in SPM12, as implemented in MATLAB R2015b. fMRI data were realigned to the first volume to correct for in-scanner head motion. Next, using the ArtRepair toolbox in SPM12, all volumes with frame-to-frame movement >1 mm or >1.5% standard deviation from the mean signal were substituted using linear interpolation from neighboring frames. After realignment and motion-correction, the fMRI data and anatomical T1-image were co-registered, followed by normalization to Montreal Neurological Institute (MNI) standard atlas and finally spatially smoothed with a 6 mm^3^ full width at half maximum (FWHM) Gaussian kernel.

For details about fMRI data exclusions at baseline we refer to our earlier paper (Gooskens et al., 2019). Of the data from 72 participants who successfully performed the fMRI stop-signal task at follow-up, thirteen datasets were excluded due to excessive head motion (>3 mm absolute movement) (ASD N = 3, OCD N = 4, TD N = 2) or replacement of more than 20% of total volumes (ASD N = 1, TD N = 3), both measured in the ArtRepair step. This resulted in 26 ASD, 16 OCD and 53 TD baseline datasets, and 21 ASD, 9 OCD and 29 TD follow-up datasets to carry forward to the fMRI analysis (see Supplement Tables S1 and S2 for sample characteristics).

At the first level, onsets of three event types (correct go-trials, successful stop-trials, failed stop-trials) were modeled using delta functions convolved with the canonical haemodynamic response function (HRF). Six motion estimation parameters were included in the model as regressors of no interest.

### 2.8 fMRI statistical analyses

Second-level random effects analyses were run for two contrasts of interest: successful stopping was investigated by contrasting successful stop trials with correct go trials (successful stop activation > go activation). Failed stopping was investigated by contrasting failed stop trials with correct go trials (failed stop activation > go activation). *fMRI sample analyses:* In the sample of children who had useable fMRI data developmental differences between diagnostic groups were assessed with LME models, using data-driven ROIs that were defined in the baseline study (Gooskens et al., 2019). For each ROI, LME models were fit with diagnostic status, time point, and age as fixed factors, and within subject dependence as a repeated measures random factor. Follow-up analyses and brain-behavior correlation analyses for the sample with data at both time points are reported in the Supplemental Material.

### 2.9 Power analysis

We used G*Power 3.1 (Faul et al., 2007) to conduct a post-hoc power analysis to estimate the sample size that would be necessary to detect between-group interaction effects, if the effects in this study were in fact real. Power analyses were run on the subsample of subjects with complete data (from both time points, N = 40). Sample size N was computed as a function of the required power level (0.80), the prespecified significance level α (0.05), and the effect size (η_p_^2^) found in the current study

## 3 Results

### 3.1 Group characteristics

*Full behavioral sample.* The characteristics of the full behavioral sample are provided in Table 1. For this sample, diagnostic groups did not differ in age, sex, or estimated IQ. Children with ASD or OCD scored higher on the compulsivity subscale and total score of the RBS-R, and on the CPRS-R compared to typically developing children (Table 2). Moreover, children with OCD scored higher on the compulsivity scale of the RBS-R than children with ASD. In each diagnostic group, drop-outs did not differ in age, IQ, symptomatology or in any of the task performance measures, compared to the children that were included in the study, indicating bias due to drop-out is unlikely.

*fMRI sample.* Diagnostic groups in the fMRI subsample (baseline: N = 95, follow-up: N = 59), did not differ in age or sex, similar to the full behavioral sample. However, IQ differed between groups at baseline, with lower scores for children with OCD than typically developing children (see Supplemental Table S1). For children with OCD, compulsive behavior on the CY-BOCS and the RBS-R compulsivity scale decreased with development, whereas for children with ASD the RBS-R total score decreased with development. Similar to the full sample, children with ASD or OCD had significantly higher scores on the compulsivity scale and total score of the RBS-R, and on the CPRS-R than TD children, in the fMRI sample (Supplemental Table S2).

### 3.2 Behavioral results

**Fig. 1:**
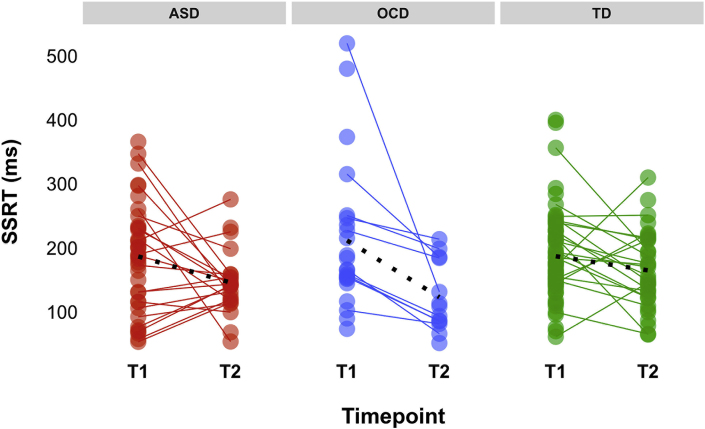
Stop-signal reaction time (SSRT) scores at baseline and follow-up for the three diagnostic groups. Black dashed line represents average slopes. SSRT decreased over development for all children, regardless of diagnosis. Abbreviations: ASD, Autism Spectrum Disorder; OCD = Obsessive-Compulsive Disorder; TD = typically developing; ms = milliseconds; T1 = timepoint 1/baseline; T2 = timepoint 2/follow-up.

Linear mixed-effects (LME) models showed a main effect of development (time 1 > time 2) [*F*_1,155_ = 6.825, *p* = .009] and an effect of age [*F*_1,122_ = 4.037, *p* = .046] on stop-signal reaction time (SSRT) in the full behavioral sample (Fig. 1, Table 2). In the fMRI sample, we found a main effect of development on SSRT only (time 1 > time 2) [*F*_1,117_ = 4.028, *p* = .047] (Fig. 1, Table S2). There were no differences in SSRT between diagnostic groups at baseline or follow-up.

There was an interaction effect between diagnostic group and development on SSD in both the full behavioral and fMRI sample (full behavioral: [*F*_2,104_ = 3.809, *p* = .022]; fMRI: [*F*_2,105_ = 3.114, *p* = .049]), where SSD increased over development for children with OCD, but not for children with ASD and TD children. In addition, we found an age effect on mean reaction time (MRT) in the full behavioral [*F*_1,137_ = 9.165, *p* = .003] and fMRI sample [*F*_1,146_ = 7.099, *p* = .009], indicating that mean reaction time decreased with age. Finally, we found an interaction effect between diagnostic group and development on MRT in the fMRI sample only [*F*_2,146_ = 5.287, *p* = .006], where reaction time decreased over development for children with OCD and TD children, but not children with ASD.

### 3.3 ROI-analysis

**Fig. 2:**
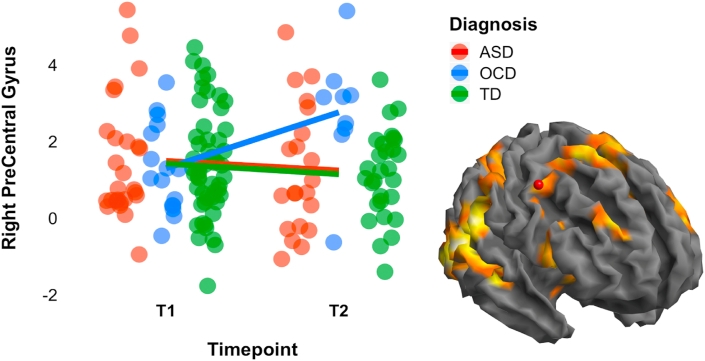
Activity in right precentral gyrus at baseline and follow-up during the fMRI stop-signal task. Lines represent average slopes. Activity increased with development for children with OCD during failed stopping. Note: the red dot in brain marks the right precentral gyrus area. Abbreviations: ASD, Autism Spectrum Disorder; OCD = Obsessive-Compulsive Disorder; TD = typically developing; ms = milliseconds; T1 = timepoint 1/baseline; T2 = timepoint 2/follow-up.

In the fMRI sample, we found an interaction between diagnostic group and development for activity in right precentral gyrus (PreC) during failed-stop trials [*F*_2, 73_ = 5.644, *p* = .005], driven by increases in activity for the OCD group over development (t (89) = −2.865, *p* = .005), while activity in other groups remained stable over development (Fig. 2).

### 3.4 Analysis of subsample with complete data at both timepoints

To confirm that no bias was introduced by participants with data at only one timepoint, we repeated the behavior- and ROI-analyses (Table S4) and performed brain-behavior correlations (Tables S5 and S6) in the subsample of participants who had complete data at both timepoints (N = 40). We could not replicate the finding of increased right precentral gyrus activity from the ROI-analysis in this subsample. Furthermore, we did not succeed in replicate findings from our baseline paper, where we found an association between increased brain activity and ADHD symptoms (Gooskens et al., 2019). A full description of characteristics of and results from this sample are provided in the Supplemental Materials.

### 3.5 Power analysis

The effect sizes (η_p_^2^) for interaction effects between groups over time were in the very small to small range (range: 0.001 to 0.134). The power analysis showed that with these effect-sizes, a minimum of 56 to more than 5000 participants would have been required to reach a power level of 0.80 to discriminate between children with ASD and OCD, and typically developing volunteers. This suggests that most differences between children with and without diagnoses in terms of task performance and brain activity were in the range of noise and unlikely to be clinically meaningful (Table S7).

## 4 Discussion

We examined the development of the behavioral and neural correlates of cognitive control in a multicenter, longitudinal study spanning a year and a half, and using a modified stop-signal task in children with ASD or OCD, and typically developing (TD) children. At baseline, children with ASD or OCD showed no changes in cognitive control or changes in brain activity in task-relevant neural networks compared to TD children (Gooskens et al., 2019). In the current study, we found the expected developmental improvement in cognitive control regardless of diagnostic group, with only subtle differences between the groups in terms of the development of task performance and brain activity. The results did not support our hypotheses on developmental changes in cognitive control and associated neural circuitry in ASD and OCD. Nor did we find support for the hypothesis that cognitive control, as assessed by the stop-signal task, is associated with repetitive behavior in these children. Rather, our findings suggest that, similar to the lack of differences at baseline, the development of cognitive control in children with ASD or OCD follows a pattern similar to that of typically developing children, at least in the age range investigated here.

As expected, we found that SSRT decreased across groups with no differences between diagnostic groups in baseline or follow-up average scores, indicating improvement in cognitive control with development. The findings are in line with previous work in typical development (Huizinga et al., 2006). We found no evidence that children with ASD or OCD showed delayed development of cognitive control over this short time. As such, these findings support the suggestion from our earlier study that any impairments in cognitive control in ASD and OCD may emerge later during development, to support findings of decreased cognitive control in adults with OCD (Chamberlain et al., 2007; Kang et al., 2013; Marzuki et al., 2020; Penadés et al., 2007; De Wit et al., 2012). This raises questions about the place of cognitive control in a mechanistic, causal cascade of repetitive behavior in these disorders.

We also did not find the hypothesized delay in the development of prefrontal brain activity in children with ASD and OCD. Rather, we found a subtle developmental difference where activity in right precentral gyrus during failed stop trials increased for children with OCD. The precentral gyrus is part of Brodmann area 6 (BA6), including pre-motor cortex and supplementary motor area (SMA), and is involved in the planning of movements. Activity in this region during response inhibition has previously been reported in healthy adults, as well as adults with OCD (Roth et al., 2007). Speculatively, this finding may suggest that, in contrast to children with ASD and TD children, children with OCD recruit precentral gyrus more for cognitive control during late childhood.

The supplemental analyses for the subsample with complete data at both timepoints showed no brain-behavior correlations in children with ASD and OCD. There was also no evidence in this subsample that either task performance or activity in frontostriatal circuitry was related to severity of or changes in repetitive behavior in children with ASD and OCD, similar to what has been found cross-sectionally using similar tasks (Ambrosino et al., 2014). We did observe a developmental increase in task performance (reflected by shorter SSRTs) that was associated with decreased activity in right middle cingulate gyrus (MCG) during successful stopping in typically developing children. However, we cannot rule out that the relatively short interval (average 1.48 years) between both assessments and the reduction in symptoms with age may have affected the lack of brain-behavior correlations in ASD and OCD. Furthermore, a post-hoc power analysis showed that vast numbers of participants (up to 5000) would have been necessary to detect between-group interaction effects over time. Even though we cannot rule out that some of our findings of no differences between groups were caused by a lack of statistical power, it must be noted that the effect sizes were mostly very small (.001 < η_p_^2^ < 0.086), with only one exception of small effect size (η_p_^2^ < 0.134), suggesting that the clinical relevance of these effects is minimal, and would continue to be so in even a more adequately powered study.

Given the problems children with ASD and OCD experience in everyday life, our observations raise the question to what extent cognitive control problems in ASD or OCD are adequately captured by commonly used cognitive control paradigms (Geurts et al., 2009). It has been suggested that findings of deficits in cognitive control are dependent on the type of task, with diagnostic differences observed more frequently on interference tasks (Adams and Jarrold, 2012; Christ et al., 2007) than stop-signal tasks (Adams and Jarrold, 2012; Chantiluke et al., 2015; Gooskens et al., 2019; Hollestein et al., 2020; Ozonoff and Strayer, 1997; Albajara Sáenz et al., 2020; Schmitt et al., 2018). Moreover, cognitive control may be affected only when particularly salient stimuli are used as stimuli (Bos et al., 2019, Bos et al., 2020, Somerville et al., 2011, Yerys et al., 2013). One important explanation for the inconsistency between paradigms and studies may be the fact that cognitive control is a multi-faceted construct, related to distinct, but overlapping brain circuits, where distinct components of cortico-striatal-thalamocortical (CSTC) circuits may be related to different types of control and repetitive behavior (Carlisi et al., 2017; Friedman and Miyake, 2004; Zhang et al., 2017; Nigg, 2017; Langen et al., 2011; Mataix-Cols et al., 2004).

The findings from our study should be considered in the context of some practical limitations and strengths. First, and inherent to longitudinal studies, there were drop-outs at follow-up which led to a modest final sample size, and may have affected our ability to find developmental effects. We addressed this issue by using two different analysis strategies: linear mixed-effects modelling allowing for missing data to reduce bias introduced by drop-outs, and a follow-up analysis by means of repeated measures ANOVA of participants with complete data at both timepoints (missing data are not permitted in this type of analysis). Furthermore, the group of children with OCD were affected relatively mildly, as assessed with the CY-BOCS, which may also have affected our ability to find developmental effects. Lastly, although this longitudinal study with two measurements already provides valuable information on the development of cognitive control, studies with three or more timepoints would permit the more precise mapping of trajectories.

To summarize, we found only subtle differences in the development of cognitive control and associated brain circuitry in children with OCD. We found no notable differences in cognitive control or brain activity in children with ASD or OCD compared to TD children. We found no evidence that cognitive control, as assessed by the stop-signal task, was associated with repetitive behavior in children with ASD and OCD. Heterogeneity of samples (including in age) and dissimilarity in task design are factors that likely contribute to the inconsistency in findings between studies. Therefore, to detect differences in these neurodevelopmental disorders it will be critical to run longitudinal studies with larger sample sizes, more than two timepoints and longer time-intervals, using similar task designs, and to adopt a dimensional approach to mapping cognitive control.

## Declaration of competing interest

The authors declare that they have no known competing financial interests or personal relationships that could have appeared to influence the work reported in this paper.
