# Supplementary material for: The development of cognitive control in children with autism spectrum disorder or obsessive-compulsive disorder: A longitudinal fMRI study
Source: Neuroimage Rep. 2021 May 30;1(2):100015. doi: 10.1016/j.ynirp.2021.100015 (PMC12172903; doi:10.1016/j.ynirp.2021.100015)
Supplement: Supplementary file 1 — Multimedia component 1 [file mmc1.docx]

**Supplemental Material to “The development of cognitive control in children with Autism Spectrum Disorder or Obsessive-Compulsive Disorder: a longitudinal fMRI study”**

Bram Gooskens^1^, Dienke J. Bos^1^, Jilly Naaijen^2,3^, Sophie E.A. Akkermans^2,3^, Anna Kaiser^4^, Sarah Hohmann^4^, Muriel M.K. Bruchhage^5^, Tobias Banaschewski^4^, Daniel Brandeis^4,6-9^, Steven C.R. Williams^5^, David J. Lythgoe^5^, Jan K. Buitelaar^3,10^, Bob Oranje^1^, Sarah Durston^1^, the TACTICS consortium

**Affiliations:**

^1^ Department of Psychiatry, Brain Center, University Medical Center Utrecht, Utrecht University, Utrecht, The Netherlands

^2^ Donders Centre for Cognitive Neuroimaging, Donders Institute for Brain, Cognition and Behaviour, Radboud University, the Netherlands

^3^ Department of Cognitive Neuroscience, Donders Institute of Brain, Cognition and Behaviour, Radboud University Medical Center, Nijmegen, The Netherlands

^4^ Department of Child and Adolescent Psychiatry and Psychotherapy, Central Institute of Mental Health, Medical Faculty, Mannheim/Heidelberg University, Mannheim, Germany

^5^ Department of Neuroimaging, King’s College London, Institute of Psychiatry, Psychology and Neuroscience, London, UK

^6^ Department of Child and Adolescent Psychiatry and Psychotherapy, Psychiatric Hospital, University of Zurich, Zurich, Switzerland

^7^ Center for Integrative Human Physiology, University of Zurich, Zurich, Switzerland

^8^ Neuroscience Center Zurich, University of Zurich, Zurich, Switzerland

^9^ ETH Zurich, Zurich, Switzerland

^10^ Karakter Child and Adolescent Psychiatry University Center, Nijmegen, The Netherlands

**Supplemental Methods**

*Behavior and ROI analysis in subsample with complete data at both timepoints (T1 & T2)*. To confirm that no bias was introduced by the participants with data at only one of the timepoints, the behavior- and ROI LME-analyses were repeated in the subsample of participants who had complete data at both timepoints (N = 40, sample characteristics are in Supplemental Table S3 and S4). To increase power, children with ASD (N = 14) and OCD (N = 6) were combined into a single group, similar to in our previous paper. Further, whole-brain main and interaction effects were explored for the two contrasts (successful/failed stopping) using the ‘flexible factorial’ module in SPM12. Subsequently, a design matrix was created that included the factor ‘subject’, the group variable ‘diagnostic status’ (ASD-OCD/control) and the within-subject factor 'time’ (timepoint 1/timepoint 2). All tests were Family-Wise Error (FWE) corrected at a p-value of .05.

*Behavior correlation analyses in subsample with complete data at both timepoints.* In order to examine tentative associations between change in task performance and change in symptom severity, we calculated delta scores for participants with complete data (time 2 – time 1) for the task performance measures and questionnaire scores (RBS-R compulsive subscale, total score; CY-BOCS total score; CPRS-R total score). Subsequently, we ran Spearman’s correlations to relate change in performance to change in symptoms. The number of administered CY-BOCS in the ASD group was too low (N = 6 at follow-up) to include in any further analyses. Additionally, a simple linear regression was calculated to explore if behavior or ROI brain measures at baseline could predict the course of repetitive behavior in children with ASD and OCD analyzed together.

As children with ASD and OCD often have symptoms of ADHD, and to replicate findings from our baseline paper (Gooskens et al., 2019), we repeated the analysis on ADHD symptomatology, where we used a median split to create two groups based on CPRS-R score (Low < 56; High > 57).

*Brain-behavior correlation analyses in subsample with complete data at both timepoints.* We assessed possible correlations between change in brain activity (whole brain and ROI) and change in task performance and symptom severity. We used the delta scores (time 2 – time 1) for the ROIs, task performance and questionnaires (RBS-R compulsive subscale, total score; CY-BOCS total score; CPRS-R total score) and calculated Spearman’s correlations. *P*-values were adjusted for multiple testing, using the Benjamini-Hochberg procedure to control the False Discovery Rate (FDR) (Benjamini & Hochberg, 1995).

**Supplemental Results**

*Behavioral effects in subsample with complete data at both timepoints.* Results from this subsample converged with those of the larger samples, as we found a main effect of development on SSRT [*F*_1,69_ = 4.383, *p* =.040, η^2^ = .065], where SSRT decreased over development. In addition, we found an age [*F*_1,36_ = 6.236, *p* = .017] and site effect [*F*_3,33_ = 4.975, *p* = .006] on MRT. We found no main effects or interaction effects of diagnostic group or development for any of the other behavioral measures (MRT, SSD, number of omissions, choice errors) in the subsample with complete data at both timepoints (Table S4).

*ROI findings in subsample with complete data at both timepoints.* The ROI finding was not replicated in this subsample (N = 40). Further, in line with our baseline results, we found no group differences in brain activation that survived whole-brain correction (*p*_FWE_ < .05). Nor were there any main effects of development, or group by development interactions in brain activity during successful and failed stopping.

*Behavioral correlations in subsample with complete data at both timepoints.*  There were no correlations between developmental change in task performance and symptom severity as assessed with questionnaires (RBS-R, CY-BOCS, CPRS-R). However, greater cognitive control (SSRT) at baseline, predicted increased repetitive behavior (total RBS-R score) at follow-up (*β* = -.060, *p* = .008) in children with ASD and OCD (F_1,18_ = 8.772, *p* = .008, *R^2^* = 3.28). Further, brain behavior correlation analysis in this subsample showed that for typically developing children, change in activity in right middle cingulate gyrus, was positively associated with the developmental change in SSRT [*r* = 0.59, *p* = .007], and negatively with change in SSD [*r* = -0.62, *p* = .005] during successful stop trials (Table S5). During failed stop-trials, change in activity in right precentral gyrus correlated negatively with change in MRT [*r* = -0.79, *p* < .001] and change in SSD [*r* = -0.79, *p* < .001] (Table S5).

*Brain-behavior correlations in subsample with complete data at both timepoints.* We found no brain-behavior correlations in children with ASD and OCD (who were combined into a single group for this analysis) (Table S6), nor did we find associations between change in task performance and change in brain activity in children with lower or higher ADHD symptoms that survived Bonferroni-correction.

|  | **ASD** | **OCD** | **TD** | **Statistical test** | **P-value** |
| --- | --- | --- | --- | --- | --- |
| n T1 | 26 | 16 | 53 |  |  |
| n T2 | 21 | 9 | 29 |  |  |
| Sex (m/f) T1 | 17/9 | 7/9 | 29/24 | χ^2^(2) = 1.936 | .380 |
| Sex (m/f) T2 | 14/7 | 5/4 | 16/13 | χ^2^(2) = 0.729 | .694 |
| Age in years T1, mean (SD) | 11.33 (1.07) | 10.92 (1.47) | 10.76 (1.15) | F_2, 92_ = 2.012 | .140 |
| Age in years T2,  Mean (SD) | 12.33 (1.56) | 12.43 (1.58) | 11.95 (1.27) | F_2, 92_ = 1.290 | .533 |
| Interval in years (SD) | 1.50 (0.42) | 1.38 (0.25) | 1.49 (0.34) | F_2, 37_ = 0.234 | .786 |
| Estimated IQ T1, mean (SD) | 108.88 (16.67) | 100.72 (13.26) | 111.93 (10.36) | K-W χ^2^ (2) = 7.999 | .018* |
| Estimated IQ T2, mean (SD) | 110.87 (14.16) | 105.82 (11.21) | 115.03 (7.72) | K-W χ^2^ (2) = 3.515 | .172 |

**Table S1.** Demographic characteristics of the fMRI sample

Abbreviations: T1 = Timepoint 1; T2 = Timepoint 2; SD = standard deviation

**Table S2.** Clinical and performance measures of the fMRI sample

|  | **T1** | **T2** | **T1** | **T2** | **T1** | **T2** | **Statistics** | | |
| --- | --- | --- | --- | --- | --- | --- | --- | --- | --- |
|  | **ASD**  ***N* = 26** | **ASD**  ***N* = 21** | **OCD**  ***N* = 16** | **OCD**  ***N* = 9** | **TD**  ***N* = 53** | **TD**  ***N* = 29** | **Time** | **Group** | **Time***  **Group** |
|  | Mean (SD) | Mean (SD) | Mean (SD) | Mean (SD) | Mean (SD) | Mean (SD) | *P*-value | *P*-value | *P*-value |
| **Questionnaires** |  |  |  |  |  |  |  |  |  |
| CY-BOCS |  |  |  |  |  |  |  |  |  |
| - Obsessions |  |  | 7.19 (5.23) | 8.13 (6.24) |  |  | .124 | n.a. | n.a. |
| - Compulsions |  |  | 10.19 (3.51) | 7.78 (5.65) |  |  | .066 | n.a. | n.a. |
| - Total score |  |  | 17.38 (7.70) | 15.00 (11.74) |  |  | .038*^a^ | n.a. | n.a. |
| RBS-Revised |  |  |  |  |  |  |  |  |  |
| - Compulsivity | 2.04 (2.65) | 2.24 (2.84) | 4.69 (3.03) | 2.67 (2.12) | 0.06 (0.31) | 0.04 (0.19) | 0.273 | < .001*^f^ | .041*^a^ |
| - Total score | 20.04 (16.04) | 17.57 (17.37) | 16.47 (11.36) | 12.00 (10.25) | 0.64 (1.30) | 0.54 (1.14) | 0.319 | < .001*^f^ | .030*^b^ |
| CPRS-Revised: Long |  |  |  |  |  |  |  |  |  |
| - Inattention | 62.85 (11.93) | 59.86 (10.16) | 57.36 (10.62) | 54.13 (8.31) | 45.63 (6.25) | 46.18 (6.19) | .388 | < .001*^f^ | n.s. |
| - Hyperactivity | 62.90 (13.16) | 61.50 (12.67) | 61.29 (10.51) | 58.00 (10.62) | 46.92 (3.78) | 45.81 (4.24) | .959 | < .001*^f^ | n.s. |
| - Total score | 64.40 (12.78) | 61.65 (11.03) | 59.93 (10.77) | 56.50 (6.89) | 45.26 (4.76) | 44.82 (4.11) | .256 | < .001*^f^ | n.s. |
| **Performance** |  |  |  |  |  |  |  |  |  |
| MRT | 561.27 (101.11) | 503.93 (67.28) | 516.08 (93.15) | 571.60 (113.23) | 516.41 (90.59) | 546.20 (56.39) | .112 | .669 | .006*^c^ |
| SSD | 370.36 (87.36) | 331.99 (79.56) | 336.01(111.96) | 417.63 (114.26) | 381.72 (144.27) | 348.30 (56.56) | .500 | .695 | .049*^d^ |
| SSRT | 165.94 (71.68) | 145.25 (51.48) | 190.72 (79.99) | 120.65 (61.30) | 188.67 (69.32) | 168.89 (63.87) | .047*^e^ | .423 | n.s. |
| Omissions | 2.02 % | 2.20 % | 2.43 % | 1.66 % | 2.24 % | 1.58 % | .934 | .865 | n.s. |
| Choice errors | 2.79 % | 3.63 % | 4.25 % | 3.56 % | 4.37 % | 3.99 % | .351 | .529 | n.s. |
| Successful Stopping | 52.4 % | 51.4 % | 51.2 % | 52.8 % | 51.41% | 51.38 % | .695 | .522 | n.s. |

Abbreviations: T1 = Timepoint 1; T2 = Timepoint 2; ASD = autism spectrum disorder; OCD = obsessive-compulsive disorder; TD = typically developing group; SD = standard deviation; m/f = male/female; ADI = Autism Diagnostic Interview; CY-BOCS = Children’s Yale-Brown Obsessive-Compulsive scale; RBS = Repetitive-Behavior scale; CPRS = Conners’ Parent Rating scale; MRT = Mean reaction time; SSD = Stop-signal delay; SSRT = Stop-signal reaction time; n.a. = not applicable; n.s. = not significant (removed from model)

^a^ Children with OCD show a decrease in compulsive behavior over development

^b^ Children with ASD show a decrease in total severity of repetitive behavior over development

^c^ Children with OCD and TD show slower reaction times over development

^d^ Children with OCD show greater SSD-latencies over development

^e^ All children show decreased SSRTs over development

^f^ ASD, OCD > TD

|  | ASD | OCD | TD | Statistical test | P-value |
| --- | --- | --- | --- | --- | --- |
| n | 14 | 6 | 20 |  |  |
| Sex (m/f) | 8/6 | 3/3 | 7/13 | χ^2^(2) = 1.703 | .202 |
| Age in years T1, mean (SD) | 11.39 (1.37) | 10.34 (1.25) | 10.43 (1.18) | F_2, 37_ = 2.783 | .075 |
| Age in years T2,  Mean (SD) | 12.89 (1.36) | 11.73 (1.47) | 11.92 (1.27) | F_2, 37_ = 2.709 | .080 |
| Interval in years (SD) | 1.50 (0.42) | 1.38 (0.25) | 1.49 (0.34) | F_2, 37_ = 0.234 | .786 |
| Estimated IQ T1, mean (SD) | 110.46 (16.99) | 100.41 (6.94) | 115.32 (7.80) | K-W χ^2^ (2) = 8.740 | .013* |
| Estimated IQ T2, mean (SD) | 108.59 (14.37) | 99.49 (7.03) | 114.87 (7.94) | F_2, 37_ = 5.191 | .010* |

**Table S3.** Sample characteristics of the subsample with complete data at both timepoints

Abbreviations: T1 = Timepoint 1; T2 = Timepoint 2; SD = standard deviation

**Table S4.** Clinical and performance measures of the subsample with complete data at both timepoints

|  | **T1** | **T2** | **T1** | **T2** | **T1** | **T2** | **Statistics** | | |
| --- | --- | --- | --- | --- | --- | --- | --- | --- | --- |
|  | **ASD**  ***N* = 14** | **ASD**  ***N* = 14** | **OCD**  ***N* = 6** | **OCD**  ***N* = 6** | **TD**  ***N* = 20** | **TD**  ***N* = 20** | **Time** | **Group** | **Time***  **Group** |
|  | Mean (SD) | Mean (SD) | Mean (SD) | Mean (SD) | Mean (SD) | Mean (SD) | *P*-value | *P*-value | *P*-value |
| **Questionnaires** |  |  |  |  |  |  |  |  |  |
| CY-BOCS |  |  |  |  |  |  |  |  |  |
| - Obsessions |  |  | 6.83 (5.78) | 6.60 (6.27) |  |  | .080 | n.a. | n.a. |
| - Compulsions |  |  | 10.17 (3.19) | 6.67 (5.43) |  |  | .022*^a^ | n.a. | n.a. |
| - Total score |  |  | 17.00 (8.39) | 12.17 (11.13) |  |  | .025*^a^ | n.a. | n.a. |
| RBS-Revised |  |  |  |  |  |  |  |  |  |
| - Compulsivity | 1.50 (2.31) | 1.64 (1.82) | 3.83 (3.49) | 2.33 (2.07) | 0.15 (0.49) | 0.05 (0.22) | 0.884 | < .001*^d^ | n.s. |
| - Total score | 18.57 (14.81) | 13.64 (11.51) | 13.83 (13.05) | 13.00 (11.31) | 0.60 (1.54) | 0.65 (1.27) | 0.251 | < .001*^d^ | .014*^b^ |
| CPRS-Revised: Long |  |  |  |  |  |  |  |  |  |
| - Inattention | 62.09 (13.09) | 61.36 (11.13) | 55.80 (12.32) | 50.80 (4.09) | 47.17 (8.10) | 47.40 (6.86) | .388 | < .001*^e^ | n.s. |
| - Hyperactivity | 61.60 (13.68) | 60.15 (14.02) | 61.60 (11.68) | 57.60 (9.69) | 47.09 (4.76) | 46.79 (4.71) | .959 | < .001*^d^ | n.s. |
| - Total score | 63.20 (13.48) | 62.08 (12.86) | 59.00 (12.35) | 54.20 (5.54) | 45.91 (4.97) | 45.68 (4.46) | .256 | < .001*^d^ | n.s. |
| **Performance** |  |  |  |  |  |  |  |  |  |
| MRT | 567.35 (67.59) | 499.18 (58.57) | 576.49 (53.45) | 551.39 (79.15) | 584.48 (91.69) | 549.42 (55.38) | .278 | .519 | n.s. |
| SSD | 384.17 (92.36) | 328.78 (85.89) | 361.73 (63.39) | 403.14 (123.76) | 345.26 (102.39) | 348.74 (58.07) | .553 | .461 | n.s. |
| SSRT | 146.78 (65.72) | 143.89 (55.34) | 178.50 (58.32) | 115.92 (70.87) | 201.85 (60.09) | 170.77 (61.36) | .040*^c^ | .053 | n.s. |
| Omissions | 2.14 % | 2.38 % | 2.92 % | 2.14 % | 2.46 % | 2.07 % | .459 | .853 | n.s. |
| Choice errors | 1.74 % | 2.44 % | 4.70 % | 4.77 % | 4.51 % | 4.12 % | .381 | .342 | n.s. |
| Successful Stopping | 52.6 % | 51.7 % | 52.78 % | 51.95 % | 51.67 % | 51.42 % | .991 | .406 | n.s. |

Abbreviations: T1 = Timepoint 1; T2 = Timepoint 2; ASD = autism spectrum disorder; OCD = obsessive-compulsive disorder; TD = typically developing group; SD = standard deviation; m/f = male/female; ADI = Autism Diagnostic Interview; CY-BOCS = Children’s Yale-Brown Obsessive-Compulsive scale; RBS = Repetitive-Behavior scale; CPRS = Conners’ Parent Rating scale, MRT = Mean reaction time; SSD = Stop-signal delay; SSRT = Stop-signal reaction time; n.a. = not applicable; n.s. = not significant (removed from model)

^a^ Children with OCD show a decrease in compulsive behavior over time

^b^ Children with ASD show a decrease in total severity of repetitive behavior over time

^c^ All children show decreased SSRTs over development

^d^ ASD, OCD > TD

^e^ ASD > TD

| **Table S5.** Spearman’s correlations between ROIs and task performance measures in typical developing children of the subsample with complete data at both timepoints | | | | | | | | | | | |
| --- | --- | --- | --- | --- | --- | --- | --- | --- | --- | --- | --- |
| **Successful Stopping** | **Left Hemisphere** | | | | | **Right Hemisphere** | | | | | |
|  | **Insula** | **MCG** | **MFG** | **SFG** | | **Insula** | | **MCG** | | **MFG** | |
| **MRT** | .16 | .27 | .028* | .07 | | .61 | | .013* | | .35 | |
| **SSD** | .21 | .50 | .19 | .06 | | .69 | | **.005**** | | .27 | |
| **SSRT** | .76 | .24 | .72 | .52 | | .92 | | **.007**** | | .030* | |
| **Failed Stopping** | **Left Hemisphere** | | | **Right Hemisphere** | | | | | | | |
|  | **Insula** | **MCG** | **MFG** | **Insula** | **PreC** | | **SMA** | | **MCG** | | **MFG** |
| **MRT** | .20 | .19 | .11 | .49 | **< .001**** | | .030* | | .047* | | .14 |
| **SSD** | .50 | .19 | .016* | .48 | **< .001**** | | .008* | | .024* | | .13 |
| **SSRT** | .68 | .019* | .014* | .89 | .012 | | .033* | | .14 | | .30 |
| Note: numbers are *p*-values; * = significant uncorrected (*p* < .05); ** = significant after Bonferroni correction (in bold font). Abbreviations: MCG = middle cingulate gyrus; MFG = middle frontal gyrus; SFG = superior frontal gyrus; PreC = precentral gyrus; SMA = supplementary motor area | | | | | | | | | | | |

| **Table S6.** Spearman’s correlations between ROIs and task performance measures in patients (children with ASD and OCD analyzed together) of the subsample with complete data at both timepoints | | | | | | | | | | | |
| --- | --- | --- | --- | --- | --- | --- | --- | --- | --- | --- | --- |
| **Successful Stopping** | **Left Hemisphere** | | | | | **Right Hemisphere** | | | | | |
|  | **Insula** | **MCG** | **MFG** | **SFG** | | **Insula** | | **MCG** | | **MFG** | |
| **MRT** | .50 | .41 | .16 | .11 | | .026* | | .57 | | .22 | |
| **SSD** | .79 | .15 | .09 | .07 | | .30 | | .14 | | .30 | |
| **SSRT** | .82 | .21 | .040* | .18 | | .91 | | .18 | | .63 | |
| **Failed Stopping** | **Left Hemisphere** | | | **Right Hemisphere** | | | | | | | |
|  | **Insula** | **MCG** | **MFG** | **Insula** | **PreC** | | **SMA** | | **MCG** | | **MFG** |
| **MRT** | .57 | .75 | .30 | .06 | .37 | | .87 | | .97 | | .16 |
| **SSD** | .47 | .17 | .82 | .030* | .09 | | .57 | | .26 | | .29 |
| **SSRT** | .60 | .19 | .95 | .13 | .24 | | .91 | | .12 | | .79 |
| Note: numbers are *p*-values, * = significant uncorrected (*p* < .05)  Abbreviations: MCG = middle cingulate gyrus; MFG = middle frontal gyrus; SFG = superior frontal gyrus; PreC = precentral gyrus; SMA = supplementary motor area | | | | | | | | | | | |

| Table. S7 Results from the post-hoc power analysis. | | | | | | |
| --- | --- | --- | --- | --- | --- | --- |
| Dependent variable | **Hemisphere** | **Contrast** | **η_p_²** | **Observed**  **effect size** | **Cohens f*** | **Estimated sample size*** |
| MRT | n.a. | n.a. | .015 | small | .12 | 520 |
| SSD | n.a. | n.a. | .029 | small | .17 | 268 |
| SSRT | n.a. | n.a. | .068 | small | .27 | 112 |
| Insula | Left | Successful | .036 | small | .19 | 216 |
| MCG | Left | Successful | .038 | small | .19 | 204 |
| MFG | Left | Successful | <.001 | small | .01 | > 5000 |
| SFG | Left | Successful | .134 | medium | .39 | 56 |
| Insula | Right | Successful | .003 | small | .05 | 2614 |
| MCG | Right | Successful | .016 | small | .12 | 486 |
| MFG | Right | Successful | <.001 | small | .01 | > 5000 |
| Insula | Left | Failed | .004 | small | .06 | 1960 |
| MCG | Left | Failed | .059 | small | .25 | 130 |
| MFG | Left | Failed | .002 | small | .04 | 3922 |
| Insula | Right | Failed | .026 | small | .16 | 298 |
| PreCG | Right | Failed | .019 | small | .13 | 410 |
| SMA | Right | Failed | .086 | small | .27 | 110 |
| MCG | Right | Failed | .026 | small | .16 | 298 |
| MFG | Right | Failed | <.001 | small | .01 | > 5000 |
| Note. * = estimated using G*Power 3.1 (Faul et al., 2009). Dependent variables include task performance measures (MRT, SSD, SSRT) and selected brain regions of interests (ROIs). Abbreviations: η_p_² = partial eta squared; MRT = mean reaction time; SSD = stop-signal delay; SSRT = stop-signal reaction time; MCG = middle cingulate gyrus; MFG = middle frontal gyrus; SFG = superior frontal gyrus; PreCG = precentral gyrus; SMA = supplementary motor area. | | | | | | |
